# Canine Coronavirus Infection Modulates the Biogenesis and Composition of Cell-Derived Extracellular Vesicles

**DOI:** 10.3390/biomedicines11030976

**Published:** 2023-03-21

**Authors:** Rachana Pandit, Ayodeji O. Ipinmoroti, Brennetta J. Crenshaw, Ting Li, Qiana L. Matthews

**Affiliations:** 1 Microbiology Program, Department of Biological Sciences, College of Science, Technology, Engineering and Mathematics, Alabama State University, Montgomery, AL 36104, USA; rachanapandit45@gmail.com (R.P.); ayodejimoroti@gmail.com (A.O.I.); bjcrenshaw0320@gmail.com (B.J.C.); 2 Department of Biological Sciences, College of Science, Technology, Engineering and Mathematics, Alabama State University, Montgomery, AL 36104, USA; tli@alasu.edu

**Keywords:** coronavirus, animal coronavirus, extracellular vesicles, immunomodulation

## Abstract

Coronavirus (CoV) has persistently become a global health concern causing various diseases in a wide variety of hosts, including humans, birds, and companion animals. However, the virus-mediated responses in animal hosts have not been studied extensively due to pathogenesis complexity and disease developments. Extracellular vesicles (EVs) are widely explored in viral infections for their intercellular communication, nanocarrier, and immunomodulatory properties. We proposed that coronavirus hijacks the host exosomal pathway and modulates the EV biogenesis, composition, and protein trafficking in the host. In the present study, Crandell–Rees feline kidney (CRFK) cells were infected with canine coronavirus (CCoV) in an exosome-free medium at the multiplicity of infection (MOI) of 400 infectious units (IFU) at various time points. The cell viability was significantly decreased over time, as determined by the 3-(4, 5-dimethylthiazol-2-yl)-2,5-diphenyltetrazolium bromide (MTT) assay. Post-infection EVs were isolated, and transmission electron microscopy (TEM) showed the presence of small EVs (sEVs) after infection. NanoSight particle tracking analysis (NTA) revealed that EV sizes averaged between 100 and 200 nm at both incubation times; however, the mean size of infection-derived EVs was significantly decreased at 48 h when compared to uninfected control EVs. Quantitative analysis of protein levels performed by dot blot scanning showed that the expression levels of ACE-2, annexin-V, flotillin-1, TLR-7, LAMP, TNF-α, caspase-1, caspase-8, and others were altered in EVs after infection. Our findings suggested that coronavirus infection impacts cell viability, modulates EV biogenesis, and alters cargo composition and protein trafficking in the host, which could impact viral progression and disease development. Future experiments with different animal CoVs will provide a detailed understanding of host EV biology in infection pathogenesis and progression. Hence, EVs could offer a diagnostic and therapeutic tool to study virus-mediated host responses that could be extended to study the interspecies jump of animal CoVs to cause infection in humans.

## 1. Introduction

The recent outbreak of severe acute respiratory syndrome coronavirus 2 (SARS-CoV-2) in 2019 has led to the ongoing coronavirus disease 2019 (COVID-19) pandemic, which is a persistent global health concern worldwide. SARS-CoV-2 is the third highly human pathogenic coronavirus (CoV) emerging after SARS-CoV in 2002 and the Middle East respiratory syndrome (MERS)-CoV in 2012. Unfortunately, this family of viruses has the potential to become a long-lasting global health crisis [1]. Coronaviruses (CoVs) are the largest family of enveloped, single-stranded (ss) RNA viruses that cause acute to severe respiratory, gastrointestinal, neurological, and other systematic diseases in a broad array of hosts, including humans and companion animals [2]. To date, seven low to high pathogenic human coronaviruses (HCoVs) that have caused several diseases in humans are well documented in the literature. Numerous pieces of evidence are documented about the origin of CoVs from zoonosis and cross-species transmission to infect humans through intermediate animals. For instance, bats are implicated as a natural reservoir host of many viruses, including CoVs, whereas cattle, palm civets, camels, and pangolins are suggested to be the most common intermediate hosts during direct or indirect animal-to-human transmission of CoVs [3,4,5]. In addition, there are several reports of companion animals being susceptible to the natural or experimental infection of animal and human CoVs. For instance, cats and dogs along with humans have been shown susceptible to feline coronavirus (FCoV), canine coronavirus (CCoV), SARS-CoV, and SARS-CoV-2 [6,7,8]. Correspondingly, cumulative studies have reported evidence of CCoV, FCoV-like CoVs, and canine–feline recombinant alphacoronavirus in human patients with pneumonia and acute respiratory symptoms, representing an apparent threat of clinical diseases in humans due to cat or dog CoVs [8,9]. Hence, there is an immediate need to extensively study the host susceptibility, cross-specificity, and tissue tropism of animal CoVs that are in close contact with humans to avoid direct or indirect transmission to humans. CoVs are characterized by the crown-shaped spike (S) projections from their virion surface. The S complexes bind to host receptors such as the host ACE2 (angiotensin converting enzyme-2) in case of the FCoV, CCoV, SARS-CoV, and SARS-CoV-2 for entering into the cell and leading to downstream pathogenesis [1,10].

Extracellular vesicles (EVs), particularly the exosome subtype, are studied extensively in viral infection of the inflammatory, lung, and respiratory tract [11,12,13,14]. These are heterogeneous groups of double-membrane nanovesicles routinely released from most cell types, normal or diseased, and can be isolated from biological fluids such as blood, plasma, saliva, urine, breast milk, and cerebrospinal fluid [15,16,17]. They are categorized into three major subtypes based on their formative processes, cellular origin, biological function, and most commonly their biogenesis: microvesicles, apoptotic bodies, and exosomes. Microvesicles are derived from the outward budding of the plasma membrane and fission of membrane vesicles from the cell surface and range between 150 and 1000 nm in diameter. Apoptotic bodies are the largest subtype of EVs, ranging between 50 and 5000 nm, and are released during apoptosis via blebbing of the plasma membrane. Exosomes are the smallest EVs, ranging between 30 and 200 nm, and are derived from intracellular inward budding of the limiting membrane of endocytic compartments from multivesicular bodies (MVBs), which are released as vesicles in the form of exosomes [12]. The exosome subtype is the most studied and has the highest biological functions among EVs subtypes [18]. Most importantly, exosomes have the ability to package different host or viral molecules such as genetic materials (e.g., DNA, mRNAs, miRNAs), proteins, lipids, receptors, growth hormones, and other signaling molecules from the parent cell and deliver these cargos into target cells via membrane fusion and/or endocytosis [11,18,19,20]. In previous studies, EVs were believed to have a role in unwanted waste disposal from the cell; however, later studies have reported their key roles in nanocarriers, intercellular communication, and immunomodulation during disease pathogenesis [11,13,18,21,22,23]. Additionally, exosomes can confer diverse functions via their packaged cargo to the target cells over shorter or longer distances. For instance, dendritic cell (DC)-derived exosomes add the additional function of antigen presentation as they incorporate different molecules such as MHC class I, MHC class II, CD80, and CD86 [16,24]. Similarly, the peripheral blood monocytes incorporating EVs secreted from hepatitis B virus (HBV)-infected cells conferred immunomodulatory effects via upregulation of programmed death 1 ligand-1 (PD-L1), a key immunoregulator, and downregulation of CD69, a leukocyte activation marker [25]. In another study, authors suggested the immunosuppressive effect and incorporation in multiple organs, including the liver, bone marrow (BM), and intestinal tract, through EVs from HBV-infected liver cells [26]. A COVID-19 study has also suggested that EVs secreted by SARS-CoV-2-infected lung epithelial cells transported viral RNA fragments along with host cell receptors to human-induced pluripotent stem cell-derived cardiomyocytes (hiPSC-CMs) [19]. Furthermore, El-Shennawy et al. indicated that circulating ACE2+ plasma exosomes from healthy donors and recovered COVID-19 patients blocked the viral S protein and cellular receptor binding, resulting in the inhibition of SARS-CoV-2 infection [27]. Hence, this mechanism of EV packaging, releasing, and transporting between cells while exerting immunomodulatory effects during infection and later contributing to the viral spread to non-infected cells has been well documented in several viral infections [11,13,28,29,30,31].

In the present study, we hypothesized that CoV hijacks the host exosomal pathway and alters EV biogenesis, composition, and protein trafficking in the host cell. We evaluated the impact of CCoV infection on the biogenesis, composition, and trafficking of Crandell–Rees feline kidney (CRFK) cell-derived extracellular vesicles. CCoVs are found in dogs, and different genotypes (I and II) of CCoVs can cause mild to severe enteric and other systemic diseases in dogs [32]. CRFK is an adherent cell line derived from feline kidney epithelial cells. It is susceptible to CCoV infection and has applications in respiratory and infectious disease research. Here, EVs ranging between 30 and 200 nm (sEVs) that are released from CRFK cells after CCoV infection are the primary focus. Our results showed the secretion of EVs after CCoV infection via the expression of classical exosomal markers. EVs were further evaluated for key immune biomarkers indicating their role in infection progression, immune modulation, and antiviral responses. In future studies, our goal is to evaluate the receptor-independent entry of CoV into host cells and the extracellular virus production in the host. We also aim to perform gene expression studies to study diverse specific gene functions and regulation after coronavirus infection. Nonetheless, EVs can augment or limit the viral infection and spread, which should be considered while studying the widespread multi-organ effect of CoVs in the host [19]. Therefore, EVs could offer a diagnostic and therapeutic tool to study virus-mediated host responses that could be extended to study the interspecies jump of animal CoVs to cause infection in humans.

## 2. Materials and Methods

### 2.1. Cell Culture of Crandell–Rees Feline Kidney (CRFK) Cells

We have selected CRFK cells as a model host system for CCoV infection in the present study as they are a recommended host for CCoV propagation based on ATCC [33]. The CRFK cells are adherent epithelial kidney cells derived from a 12-week-old female cat *(Felis catus)* and are susceptible to CCoV infection. [33,34]. They were obtained from American Type Culture Collection (ATCC, Manassas, VA, USA). The cells (ATCC CCL-94, cell passage number 182) were cultured in a complete growth medium, namely ATCC-formulated Eagle’s Minimum Media (EMEM) (Fisher Scientific, Grand Island, NY, USA) with L-glutamine supplemented with 10% horse serum (Fisher Scientific, Grand Island, NY, USA), 1% penicillin/streptomycin (Fisher Scientific, Grand Island, NY, USA), and 0.2% amphotericin B (0.5 ug/mL) (Fisher Scientific, Grand Island, NY, USA). The cells were maintained in a 37 °C incubator supplemented with 5% CO_2_ to approximately 70–80% confluency. For virus infection, exosome-free media were prepared with 2% exosome-depleted HS as per the protocol in the laboratory.

### 2.2. Viral Stock

Canine coronavirus (TN449 strain, ATCC VR-2068) stock was obtained from ATCC (Manassas, VA, USA). The final concentration of obtained viral stock was 8.9 × 10^3^ TCID_50_/mL in 6 days in CRFK cells at 35 °C with 5% CO_2_. The required multiplicity of infection (MOI) for virus infection was calculated via a 10-day infection cycle and observation for viral plagues and cytopathic effect (CPE).

### 2.3. In Vitro Infection of CRFK Cells

After cell densities reached 70–80% confluency, the viability of the cells was examined via trypan blue assay. The cells were trypsinized, centrifuged, resuspended, and counted using trypsin, 0.4% trypan blue solution, and a Countess cell counter (Invitrogen, Waltham, MA, USA). CRFK cells were seeded at a density of 5 × 10^5^ cells in each cell culture dish and incubated overnight at 37 °C in a 5% CO_2_ incubator before infection. Overnight cell-free media were discarded and 3 mL of 2% exosome-free medium was added to each dish for infection experiments. Uninfected control dishes were incubated as they were after adding exosome-free media, whereas infection dishes were infected with CCoV at MOI of 400 infectious units (IFU). Plates were incubated for 48 h and 72 h in independent experiments. The cell-free medium from each control and infection dish was collected independently post-incubation and stored at −80 °C for further EV isolation.

### 2.4. Cell Lysate

The post-incubation cells were collected via scraping after adding 1–2 mL of 1X phosphate-buffered saline (PBS) to the dish and leaving for 1–2 min. The collected cells in 1X PBS were centrifuged to collect cell pellets, and isolated cell pellets were stored at −80 °C until further cell lysate experiments.

### 2.5. 3-(4, 5-dimethylthiazo-1-2yl)-2,5-diphenyltetrazolium Bromide (MTT) Assay

The viability of CRFK cells was assessed after CCoV infection using the MTT assay. The MTT assay is a colorimetric assay for assessing cell metabolic activity as an indicator of cell viability and cytotoxicity. For this assay, 1 × 10^4^ cells were seeded in triplicate in a complete medium independently in 96-well tissue culture plates. The cells were incubated overnight at 37 °C and 5% CO_2_. The following day, cell-free media were replaced with exosome-free media in each well. The control wells remained as they were; however, the infection wells were infected with CCoV at MOI of 400 IFU which were further incubated for 48 h and 72 h. Post-incubation, 50 µL of 5 mg/mL MTT in 1X PBS was added to each well and incubated for 3–4 h in a 37 °C and 5% CO_2_ incubator. A 100 µL stop solution was added to each well post-incubation. The absorbance was then read at 570 nm, and the standard curve was plotted to evaluate the cell viability of CRFK cells after CCoV infection. Each sample was evaluated in triplicate.

### 2.6. Isolation and Purification of CRFK-Derived EVs

The CRFK-derived EVs were isolated and purified from EMEM exosome-free cell culture media as described previously in [30]. Previously collected cell-free media were spun down at 1300 revolutions per minute (rpm) for 10 min using an Allegra X-14R Centrifuge (Beckman Coulter, Brea, CA, USA). The supernatant was further spun down at 3900 rpm for 10 min and filtered through a 0.22 µm pore size filter. The filtrate was transferred to an ultracentrifuge tube, and the remaining tube was filled with 6–10 mL of 1X PBS to avoid sample spillage during ultracentrifugation. The mixture was then centrifuged at 10,800 rpm for 45 min at 4 °C in an SW41T1 swinging rotor using a Beckman Coulter Optima L-70K ultracentrifuge. The supernatant was further centrifuged at 32,000 rpm for 70 min at 4 °C in the same L-70K ultracentrifuge. Finally, the supernatant was discarded, and approximately 500 µL of resuspended EV pellets was collected from each tube from below the meniscus of each centrifuge tube. The protease inhibitor (Halt Protease Inhibitor Single Use Cocktail, Thermo Scientific) was added to the collected EVs at the concentration of 10 µL/mL for preventing rapid protein degradation. The isolated EVs were stored at −80 °C for future experimentation.

### 2.7. Total DNA and RNA Extraction

The total DNA and RNA of isolated EVs were extracted using the TRIzol Reagent (Invitrogen) for total DNA and RNA precipitation. Prior to the TRIzol procedure, 5 μg EV samples were pretreated with 1 unit (U) of RNase-free DNAase I and 1 U of micrococcal nuclease (MNase) (Thermo Scientific) for DNA and RNA extraction, respectively. For total RNA, EV samples were incubated with 1% Triton-X-100 on ice for 30 min before Mnase treatment. Samples were treated with Mnase for 15 min at 37 °C followed by RNA isolation by TRIzol extraction method as described in procedure guidelines [35]. For total DNA, EV samples were incubated with RNase-free DNase I in a water bath at 37 °C for 30 min followed by 50 mM EDTA treatment at 65 °C for 10 min and proceeded to DNA isolation by TRIzol method. Total levels of DNA and RNA in CRFK-derived control EVs and infected EVs were quantified using NanoDrop One (Thermo Scientific) and stored at −80 °C for future experimentation.

### 2.8. Total Protein Quantitation

The total amount of isolated EV proteins was quantitated via the bicinchoninic acid (BCA) protein quantification method (acidified Coomassie Brilliant Blue G-250; Bio-Rad Laboratories). Five microliters of standards (0, 0.2, 0.4, 0.8, and 1.6 µg/µL of bovine serum albumin (BSA)) and EV samples were added in triplicate in a 96-well tissue culture plate. Then, 25 µL of protein reagent A and 200 µL of protein reagent B were added to each well, and the plate was placed in aluminum wrap in a shaker for 10 min. The absorbance at 595 nm was read, and the standard curve was plotted to determine the exact concentration of total protein in isolated EVs.

### 2.9. NanoSight Tracking Analysis (NTA)

The concentration (particles per mL) and size distribution (nm) of isolated EVs were analyzed using NTA. NTA analyzes the size of the particle in fluids based on the rate of Brownian motion to dynamic light scattering (DLS). The diluted EV samples (1:75 in microbial cell culture grade water) were injected into the machine sample chamber of the ZetaViewR Particle Tracking Analyzer, and the mean values (concentration and size) of particles (mean ± standard deviation of the mean values) were recorded and analyzed in 11 separate locations for each sample using the ZetaViewR Analyze (version 8.50.14 SP7) software.

### 2.10. Transmission Electron Microscopy (TEM)

The varying size and morphology of isolated EVs were visualized through TEM analysis. The EV samples were fixed with 2.5% glutaraldehyde (1:1) and loaded onto an EM grid and then incubated for 1 min at room temperature (RT). After incubation, the samples were immediately stained with filtered uranyl acetate (UA) solution and then observed under TEM (Tecnai 120 kV FEI, Hillsboro, OR, USA) as compared to the negatively stained grids. Digital images of EVs were captured using a BioSprint 29 CCD Camera (AMT Imaging, Woburn, MA, USA).

### 2.11. Dot Blot Analysis

The expression of specific protein markers associated with EVs and cell lysate was evaluated via dot blot analysis. Cell lysates were prepared by adding 400 µL of 1X lysis buffer in the previously collected cell pellet which was centrifuged to collect supernatant as a lysate. Correspondingly, 5 µg of EV proteins or cell lysate were mixed with 1X reducing loading buffer (1:1) and boiled for 10 min in a heating block at 95 °C. The mixtures were dotted on a nitrocellulose membrane, air dried, and blocked for nonspecific binding at RT for 30–45 min with 5% non-fat dry milk prepared in 0.2% Tween-20 and 1X Tris-buffered saline (TBST). After blocking, the membranes were washed three times in wash buffer (TBST) for 10 min each and incubated overnight at 4 °C with primary antibodies of CD9 (1:500 dilution, Fisher Scientific, Grand Island, NY, USA), CD63 (1:500 dilution, Santa Cruz Biotechnology, Dallas, TX, USA), cleaved Asp210 caspase-1, caspase-8 (1:500, Invitrogen, Waltham, MA, USA), cleaved caspase-3 (1:500, RD Systems, Minneapolis, MN, USA), HspPB8-13B6 (Hsp22), canine Hsp70 (1:500, Invitrogen, Waltham, MA, USA), canine Hsp90 (1:500, Novus Biologicals, Englewood, CO, USA), Hsp100 (1:500, DSHB, Iowa City, IA, USA), anti-TLR3 (1:500, Abnova Taipei City, Taiwan), TLR6 and 7 (1:500, Invitrogen, Waltham, MA, USA), Rab5 (1:500, Invitogen, Waltham, MA, USA), Rab7 (1:500, Fisher Scientific, Grand Island, NY, USA), canine anti-Rab11 (1:500, Novus Biologicals, Englewood, CO, USA), canine anti-Rab35 (1:500, Millipore Sigma, Burlington, MA, USA), JLA20 (1:500, DSHB, Iowa City, IA, USA), IL-1β (1: 500, Bioss Antibodies Inc., Woburn, MA, USA), IL-6 (1:500, DHSB, Iowa City, IA, USA), IRF 6 and 8 (1:500, DSHB, Iowa City, IA, USA), TGIF-1, TGIF-2 (1:500, DHSB, Iowa City, IA, USA), mCCL22 (1:500, RD Systems, Minneapolis, MN, USA), NFkB (1:500, Invitogen, Waltham, MA, USA), iNOS (1:500, RD Systems, Minneapolis, MN, USA), LAMP-Human (1:500, DSHB, Iowa City, IA, USA), TNF-α (1:500, Bioss Antibodies Inc., MA, USA), a6F, rr1, CXCL2 (1:500, DSHB, Iowa City, IA, USA), TSG101 (1:500, Fisher Scientific, Grand Island, NY, USA), Alix (1:500, Fisher Scientific, Grand Island, NY, USA), anti-flotillin-1 (1:500, BD Bioscience, Franklin Lakes, NJ, USA), HBB2 (1:500, DHSB, Iowa City, IA, USA), annexin-V (Fisher Scientific, Grand Island, NY, USA), anti-canine coronavirus pAB (1:500, Abnova, Taipei City, Taiwan), HERMES-1/CD44 (1:500, DSHB, Iowa City, IA, USA), H5C6/LAMP-3 (1:500, DSHB, Iowa City, IA, USA), syncytin-1 (1:500, Bioss Antibodies Inc., Woburn, MA, USA), and so on. The membranes were washed three times prior to adding horseradish peroxidase (HRP)-conjugated secondary antibodies (goat anti-mouse (1:500–1:2000 dilution, Fisher Scientific, Grand Island, NY, USA), goat anti-rabbit (1:500–1:2000 dilution, Novus Biologicals LLC, Englewood, CO, USA), or goat anti-rat (1:500–1:2000 dilution, Fisher Scientific, Grand Island, NY, USA)) in blocking solution and incubated for 90–120 min in a shaker at RT. The membranes were washed three times for 10 min each. The specific protein signals were developed using Super Signal West Femto Maximum Sensitivity Substrate (Invitrogen, Waltham, MA, USA) and imaged via a Bio-Rad ChemiDoc TM XRS system (Bio-Rad Laboratories, Hercules, CA, USA).

### 2.12. Sodium Dodecyl Sulfate–Polyacrylamide Gel Electrophoresis and Western Blot Analysis (SDS-PAGE)

The expression of specific proteins associated with EVs and cell lysate were further evaluated via SDS-PAGE/Western blot analyses. Thirty-two micrograms of EV proteins or cell lysate was mixed with reducing loading buffer (1:1) and boiled for 10 min in a heating block at 95 °C. Samples were loaded in a 4–20% 1.5 mm Bio-Rad precast gel and ran at 100 V followed by transfer to a polyvinylidene difluoride (PVDF) membrane in a transfer chamber at 45 mA overnight. The membrane was blocked for 30–45 min at RT in a blocking solution (5% non-fat dry milk in 0.2% Tween-20 and 1X TBS). After blocking, membranes were washed three times for 10 min each and incubated overnight with the primary antibodies such as anti-TSG101 (1:1000 dilution), anti-flotillin-1 (1:1000 dilution), HBB2 (1:1000 dilution), and anti-syncytin-1 (1:1000 dilution). The following day, each membrane was washed and incubated with HRP-conjugated secondary antibodies (goat anti-mouse, goat anti-rabbit, or goat anti-rat) in a blocking solution for 90–120 min in a shaker at RT. The protein signals were developed using Super Signal West Femto Maximum Sensitivity Substrate (Invitrogen, Waltham, MA, USA) and imaged via a Bio-Rad ChemiDoc TM XRS system (Bio-Rad Laboratories, Hercules, CA, USA).

### 2.13. Statistical Analysis

Statistical analyses were performed using t-test and one-way analysis of variance (ANOVA) with Tukey post hoc analysis on obtained data points using Bio-Rad imaging program (Hercules, CA, USA) and GraphPad, Version 5 (San Diego, CA, USA) software. Dot blot images were imported into the Bio-Rad imaging program, and obtained intensity density of each dot was plotted in the GraphPad Version 5 software. Statistical significance is indicated by the mean ± standard deviation (SD) and is defined as *p* ≤ 0.05 (*), *p* ≤ 0.01 (**), *p* ≤ 0.001 (***), and *p* ≤ 0.0001 (****).

## 3. Results

### 3.1. CRFK Cell Viability after Coronavirus Infection

**Figure 1:**
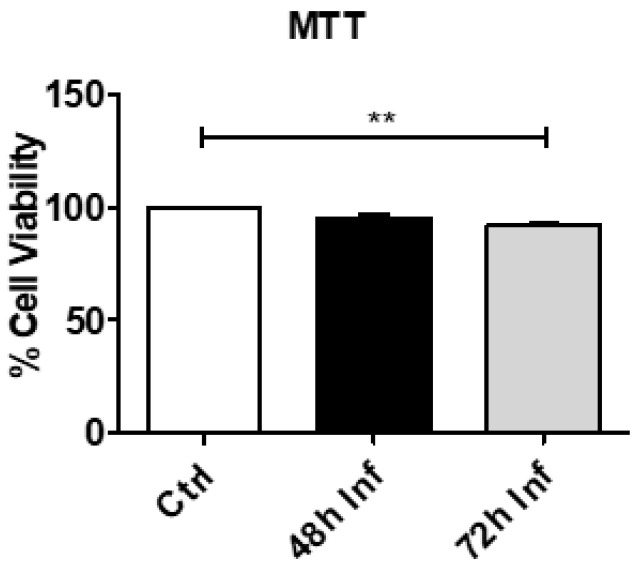
Crandell–Rees feline kidney (CRFK) cell viability after canine coronavirus (CCoV) infection. CRFK cells were infected with CCoV at MOI of 400 infectious units (IFU) in an exosome-free medium and incubated for 48 h and 72 h; post-incubation cells were further incubated with 3-(4, 5-dimethylthiazol-2-yl)-2,5-diphenyltetrazolium bromide (MTT) dye solution at 37 °C for 3–4 h and absorbance was read at 570 nm. Statistical analysis of obtained data points was performed using one-way analysis of variance (ANOVA) with Tukey post hoc analysis. Statistical significance is indicated by the mean ± standard deviation (SD) and is defined as *p* ≤ 0.01 (**).

The optimal MOI required for CCoV infection was determined via a 10 days infection assay in which CRFK cells were infected with different MOIs (10 IFU, 100 IFU, 250 IFU, and 500 IFU) of CCoV and observed for CPE every 24 h. CPE refers to the morphological changes in the host cells due to viral infections, and a few common examples of this effect include rounding of infected cells, fusion with adjacent cells, and the appearance of nuclear or cytoplasmic inclusion bodies [36]. No CPE was observed with MOIs of 10 IFU and 100 IFU for up to 10 days and 7 days post-infection, respectively; however, with MOIs of 250 IFU and 500 IFU, CPE was observed in the CRFK cells after 4 days and 2 days post-infection, respectively. Hence, we selected MOI of 400 IFU (between 250 IFU and 1000 IFU) for further experimentation at 48 h and 72 h incubation time points to simulate natural infection, based on the CPE assay. The CRFK cells were then infected with CCoV at MOI of 400 IFU in an exosome-free medium and incubated for 48 h and 72 h. The cell morphology was examined through bright-field microscopy which showed a lower number of cells with increasing incubation time with the virus (Supplementary Figure S1). The MTT assay also revealed a significant reduction in viable cells with increased incubation time after virus infection (Figure 1). At 48 h, cell viability was reduced by approximately 5%, and at 72 h, it was significantly reduced by approximately 9% (** *p* = 0.006) when compared to the uninfected control cells. Our result indicated that CCoV infection significantly caused cell death with increased incubation time in CRFK cells.

### 3.2. CRFK-Derived EV Morphology after Coronavirus Infection

**Figure 2:**
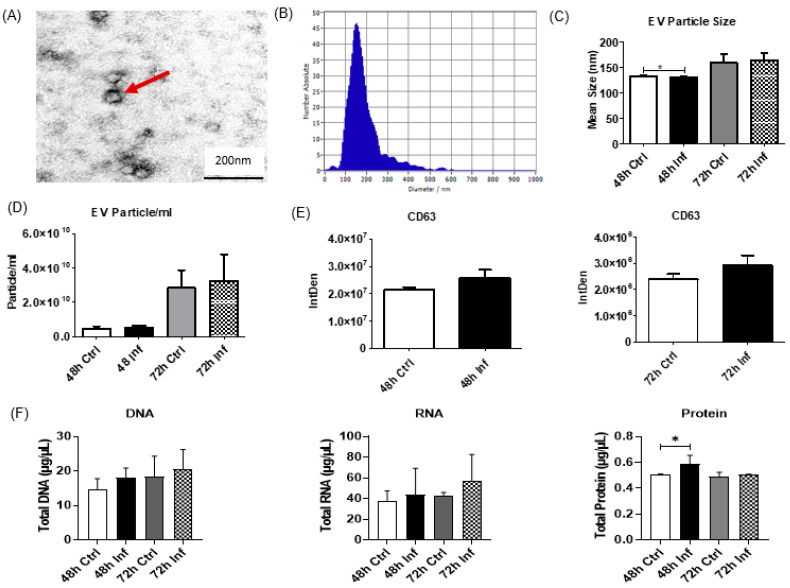
CRFK-derived extracellular vesicle (EV) characterization after CCoV infection. The morphology, size distribution (nm), and concentration (particles per mL) of CRFK-derived control EVs and infection EVs after CCoV infection were characterized. (**A**) transmission electron microscopy (TEM) images showing morphologies of CRFK-derived control sEVs at 48 h (small EVs indicated by red arrow); (**B**) nanosight particle tracking analysis (NTA) showing size distribution pattern of CRFK-derived EVs; (**C**) mean particle size; (**D**) particle concentration per mL, at 48 h and 72 h; (**E**) graphs showing densitometric analysis of dot blot (Supplementary Figure S2A) of classical EV biomarker, cluster of differentiation (CD)63, via Bio-Rad imaging program and GraphPad Version 5 software in isolated EVs at 48 h and 72 h; (**F**) total DNA, RNA, and protein content of CCoV-infected CRFK-derived control and infected EVs at 48 h and 72 h. Statistical analysis of obtained data points was performed using one-way ANOVA with Tukey post hoc analysis. Statistical significance is indicated by the mean ± SD and is defined as *p* ≤ 0.05 (*).

In the present study, CRFK cells were infected with CCoV at MOI of 400 IFU in an exosome-free medium and incubated for 48 h and 72 h before EVs were isolated from cell-free supernatant and purified via a series of high-speed ultracentrifugation steps. The isolated EVs were then evaluated for morphology, size distribution (nm), and concentration (particles per mL) via TEM and NTA. The TEM images showed the morphological presence of sEVs in both control and infected EVs after viral infection at both time points. Figure 2A is a representative TEM image of EVs derived from CRFK-CCoV infection at 30,000× magnifications at 48 h post-infection (sEV indicated by red arrow), confirming the presence of sEVs in the preparation. NTA was further performed to determine the size distribution and concentration (particles per mL) of isolated EVs and revealed that EV size in the preparation averages between 100 and 200 nm at 48 h post-incubation (Figure 2B). However, there was a significant decrease in particle size of EVs after virus infection compared to control EVs at 48 h. Control EVs have a mean diameter of 133.6 ± 0.318 nm (number of replicates (*N*) = 5, * *p* = 0.01) and the infection-derived EVs have a mean diameter size of 131.3 ± 0.684 nm at 48 h (Figure 2C). However, EV concentration (particles per mL) was relatively increased over time after CCoV infection as compared to control EVs (Figure 2D). The densitometric analysis and statistical analysis of dot blot images (Supplementary Figure S2A) of the classical EV biomarker, cluster of differentiation (CD)63, via Bio-Rad imaging program and GraphPad software, respectively, also confirmed the presence of sEVs in the preparation after viral infection (Figure 2E). Furthermore, total DNA, RNA, and protein levels were quantified in EV samples. The total EV protein content of infection EVs was significantly higher at 48 h after virus infection, while total DNA and RNA levels were significantly unchanged between infection EVs and control EVs at relative time points (Figure 2F).

### 3.3. Host Receptor Expression for Coronavirus Infection

**Figure 3:**
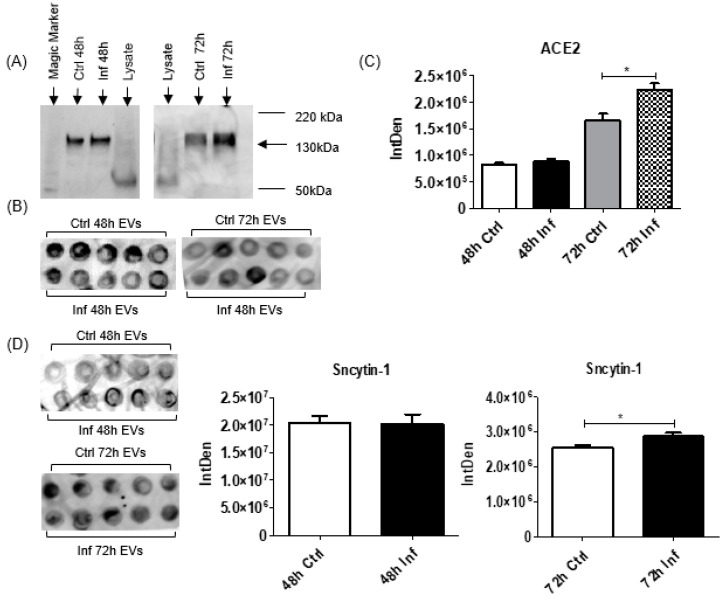
Host receptor and virus-specific protein expression levels after CCoV infection. Schematic (**A**) western blot and (**B**) dot blot showing expression of coronavirus host receptor, angiotensin-converting enzyme (ACE2), at 48 h and 72 h in the control and infection EVs; (**C**) graphs showing densitometric analysis of dot blot of ACE2 via Bio-Rad imaging program and GraphPad Version 5 software; (**D**) schematic dot blot images and densitometric graph for retroviral protein, syncytin-1, via Bio-Rad imaging program and GraphPad Version 5 software in isolated EVs at 48 h and 72 h. Statistical analysis of obtained data points was performed using one-way ANOVA with Tukey post hoc analysis. Statistical significance is indicated by the mean ± SD and is defined as *p* ≤ 0.05 (*).

ACE2, type 1 integral transmembrane carboxypeptidase protein, is a well-known cell receptor for SARS-CoV and SASR-CoV-2 entry and spread in the host. The S protein for these viruses binds to ACE2 in the host cells during entry and subsequent pathogenesis [16]. ACE2 is expressed abundantly in pulmonary and extrapulmonary cell types including lung, gut, kidney, cardiac, renal, intestinal, and endothelial cells, skin, and other organs [37]. It is primarily involved in the regulation of the renin–angiotensin system, blood pressure, and integrin signaling [38]. In the present study, ACE2 was expressed in the isolated EVs from both control CRFK cells and after CoV infection at both time points. The expression was evaluated via Western blot (Figure 3A) and dot blot (Figure 3B). The Western blot analysis of ACE2 showed gel bands at around 130 kilodaltons (kDa) for ACE2 protein which is consistent with molecular weight for native ACE2, i.e., 110–145 kDa in the literature (Figure 3A) [39,40]. The densitometry analysis of dot blot image of ACE2 via Bio-Rad imaging program and GraphPad Version 5 software showed a significantly elevated expression of ACE2 in infection EVs after virus infection (* *p* = 0.01) at 72 h when compared with EVs from uninfected control (Figure 3C). Hence, our result confirmed the expression of the CoV host receptor in the EVs isolated from CRFK control cells and after CCoV infection.

### 3.4. Virus-Related Protein Expression after Coronavirus Infection

The isolated EV cargo was evaluated for virus-related proteins to observe if CoV-specific proteins could be packaged in the EVs or not. Syncytin-1, an endogenous retroviral envelop (*env*) protein, was found to be packaged in a significantly higher amount at 72 h in EVs after viral infection (* *p* = 0.02) from the densitometric analysis of dot blot of syncytin-1 expression via Bio-Rad imaging program and GraphPad software (Figure 3D). Syncitin-1 is a fusogenic retroviral glycoprotein, and its expression represents cell fusion, exosome uptake, and internalization in host cells [39].

### 3.5. EV Biogenesis Protein Expression after Coronavirus Infection

**Figure 4:**
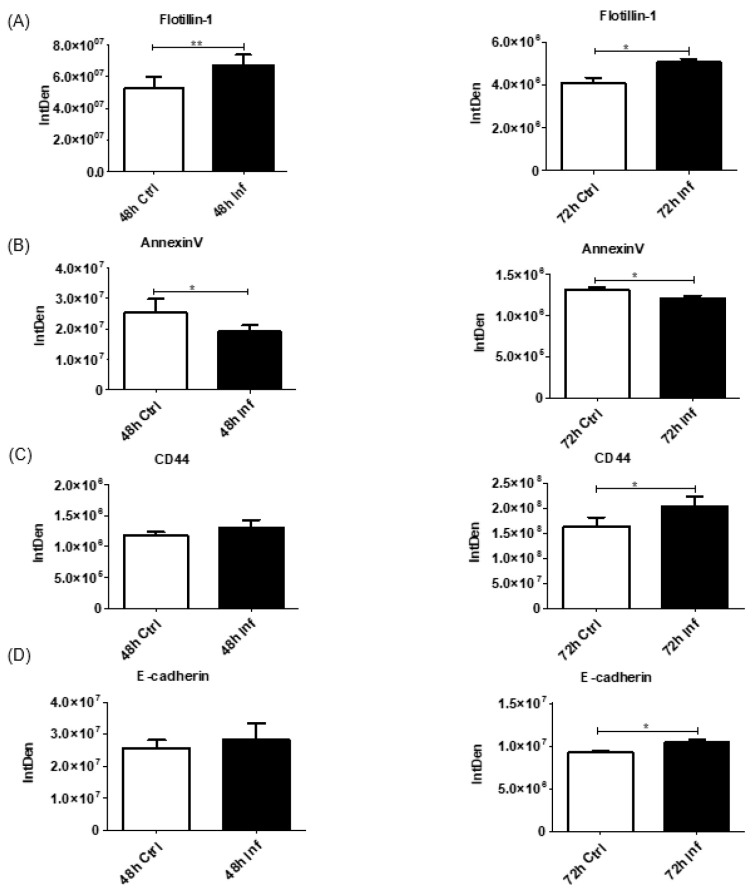
The effect of CCoV infection on EV biogenesis and membrane proteins. Graphs showing densitometric analysis of dot blot of (**A**) flotillin-1 at 48 h and 72 h (Supplementary Figure S2B), (**B**) biogenesis protein annexin-V at 48 h and 72 h (Supplementary Figure S2C), (**C**) multifunctional transmembrane receptor CD44 at 48 h and 72 h (Supplementary Figure S2D), and (**D**) canine-specific adhesion molecule E-cadherin at 48 h and 72 h (Supplementary Figure S2E), in isolated control and infection EVs. Statistical analysis of obtained data points was performed using one-way ANOVA with Tukey post hoc analysis. Statistical significance is indicated by the mean ± SD and is defined as *p* ≤ 0.05 (*) and *p* ≤ 0.01 (**).

Several membrane trafficking proteins were evaluated for their expression via dot blot in isolated EVs after CCoV infection. Flotillin-1, a membrane trafficking and biogenesis molecule, was found in significantly elevated amounts at both time points in the EVs after viral infection (* *p* = 0.02 and * *p* = 0.05, respectively) (Figure 4A) when dot blot of flotillin-1 in control and infection EVs (Supplementary Figure S2B) was analyzed via Bio-Rad imaging program and GraphPad. Flotillin is a lipid raft protein and facilitates the endocytosis, signal transduction, and biogenesis of the exosome subtype [41]. Another membrane trafficking molecule, annexin-V, was also evaluated (Supplementary Figure S2C), and we observed a significantly reduced expression at both 48 h and 72 h in infection-derived EVs when compared to control-derived EVs (* *p* = 0.02 and * *p* = 0.05, respectively) (Figure 4B). The annexin protein family can be shuttled between the cells via exosomes and from shed microvesicles and plays a major role in cell signaling, remodeling, and adaptive immune responses, among others [42]. Our result revealed that CoV infection modulates EV biogenesis and trafficking proteins in the CRFK cells.

### 3.6. Membrane Proteins in EV Cargo after Coronavirus Infection

We measured the expression of several transmembrane glycoproteins and adhesion molecules in CRFK-derived EVs after CCoV infection. CD44, a multifunctional transmembrane receptor for many growth factors and cytokines, is involved in cell–cell interactions including adhesion, proliferation, hematopoiesis, and lymphocyte activation and extravasation. It is a major receptor for hyaluronic acid, and its signaling is important in diseases such as inflammation, cancer, arthritis, and viral diseases [43]. When its expression within control and infection EVs (Supplementary Figure S2D) was measured, CD44 was found to be significantly upregulated at 72 h in infection-derived EVs compared to control-derived EVs (* *p* = 0.01) (Figure 4C), which indicates upregulated EV uptake in host cells after CCoV infection. We further examined a canine-specific cell–cell adhesion molecule, E-cadherin, and observed significantly elevated expression at 72 h in infection-derived EVs as compared to control-derived EVs (* *p* = 0.02) (Supplementary Figure S2E and Figure 4D, respectively). Our results showed that CCoV infection of CRFK cells alters membrane protein expression and packaging in the EVs.

### 3.7. Pathogen Recognition and Proinflammatory Responses after Coronavirus Infection

**Figure 5:**
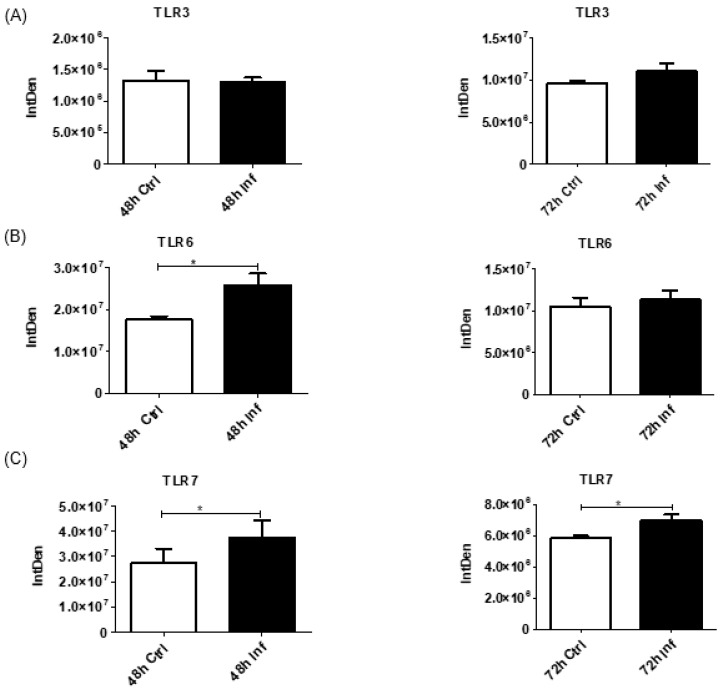
Activation of pathogen recognition molecules in response to CCoV infection. Graphs showing densitometric analysis of dot blot of (**A**) double-stranded RNA sensor TLR3 level at 48 h and 72 h (Supplementary Figure S2F), (**B**) lipopolysaccharide sensor TLR6 level at 48 h and 72 h (Supplementary Figure S2G), and (**C**) RNA sensor TLR7 level at 48 h and 72 h (Supplementary Figure S2H), via Bio-Rad imaging program and GraphPad Version 5 software in isolated control and infection-derived EVs. Statistical analysis of obtained data points was performed using one-way ANOVA with Tukey post hoc analysis. Statistical significance is indicated by the mean ± SD and is defined as *p* ≤ 0.05 (*).

Different toll-like receptors (TLRs) were evaluated in the CRFK-derived EVs in response to CCoV infection. TLRs function in viral particle recognition and innate immunity activation via secretion of proinflammatory cytokines such as interleukin (IL)-1, IL-6, tumor necrosis factor-alpha (TNF-α), interferon (INF), and INF-regulating factors (IRFs) [44,45]. While TLR3, the double-stranded RNA (dsRNA) sensor that localizes in the endosomal surface, was expressed in control and infection EVs at both times (Supplementary Figure S2F and Figure 5A, respectively), TLR6 which recognizes lipopolysaccharide was significantly upregulated at 48 h in the infection-derived EVs compared to the control-derived EVs (* *p* = 0.03) (Supplementary Figure S2G and Figure 5B, respectively). Similarly, the expression of TLR7, the single-stranded RNA (ssRNA) sensor, was significantly increased at both 48 h and 72 h in EVs after virus infection (* *p* = 0.03 and * *p* = 0.03, respectively), as shown in Supplementary Figure S2H and Figure 5C, respectively. TLR3 and TLR6 participate in the activation of myeloid differentiation primary response 88 (MyD88), and TLR7/8 activates the nuclear factor-kB (NF-kβ). Collectively, TLRs regulate the immune and inflammatory response-related gene expression levels [46]. Hence, our result suggests that TLRs regulate the expression of inflammatory and immune response-related biomarkers in the EVs during CCoV infection.

### 3.8. Stress Response to Coronavirus Infection

**Figure 6:**
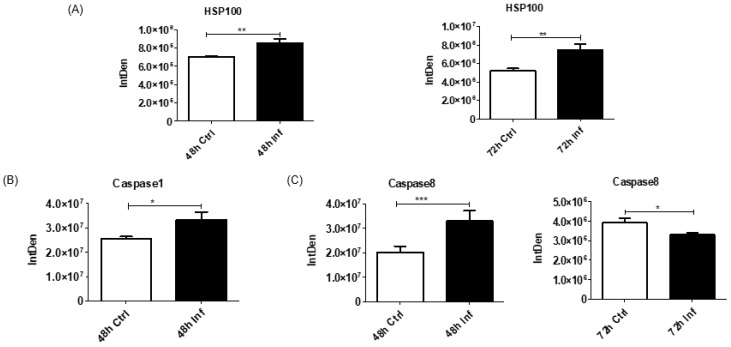
The effect of CCoV infection on heat shock protein and caspases. Graphs showing densitometric analysis of dot blot of (**A**) heat shock protein HSP100 (Supplementary Figure S2I) at 48 h and 72 h, (**B**) caspase-1 (Supplementary Figure S2J) level at 48 h, and (**C**) caspase-8 level (Supplementary Figure S2K) at 48 h and 72 h, in isolated control and infection-derived EVs. Statistical analysis of obtained data points was performed using one-way ANOVA with Tukey post hoc analysis. Statistical significance is indicated by the mean ± SD and is defined as *p* ≤ 0.05 (*), *p* ≤ 0.01 (**) and *p* ≤ 0.001 (***).

Heat shock proteins (HSPs) are constitutively expressed molecular chaperones that are involved in protein unfolding, misfolding, or aggregation during the cell-division cycle required for cell growth and development. They check different processes such as host entry, replication, and survival during pathogen infection. Hence, their expression is prominently induced during stressful conditions such as bacterial infections, viral infections, tumors, injury, temperature, nutrient deficiency, oxygen radicals, and other stimuli [47,48]. In the present study, HSP100 was significantly expressed at a higher level at both 48 h and 72 h in infection-derived EVs after CCoV infection when compared to control-derived EVs (** *p* = 0.009 and ** *p* = 0.009, respectively) (Supplementary Figure S2I and Figure 6A, respectively) while HSP70 (Supplementary Figure S3G) and HSP90 (Supplementary Figure S3H) were significantly unchanged between the infection EVs and control EVs at relative time points.

### 3.9. Apoptotic Activation in Response to Coronavirus Infection

To examine the EV cargo for activation of apoptotic pathways after CCoV infection, we evaluated different caspases in the control EVs and EVs after viral infection. Caspases are innate immunity modulators of cell death in response to various stimuli such as infections, injury, and others. Virus infection may induce or block cell death as a host defense mechanism to eliminate viral-infected cells or to facilitate replication in the host. Here, the expression of an inflammatory caspase, caspase-1, was significantly upregulated at 48 h (* *p* = 0.049) (Supplementary Figure S2J and Figure 6B, respectively), and the level of an extrinsic apoptosis marker, caspase-8, was significantly elevated at 48 h but reduced at 72 h (*** *p* = 0.0004 and * *p* = 0.04, respectively) in the infection-derived EVs when compared to control-derived EVs (Supplementary Figure S2K and Figure 6C, respectively). Hence, our finding showed that CCoV infection of CRFK cells modulates major caspase family proteins, and the apoptotic pathways could be mediated via caspase-1 and caspase-8 in CoV infection.

### 3.10. Immune Responses to Coronavirus Infection

**Figure 7:**
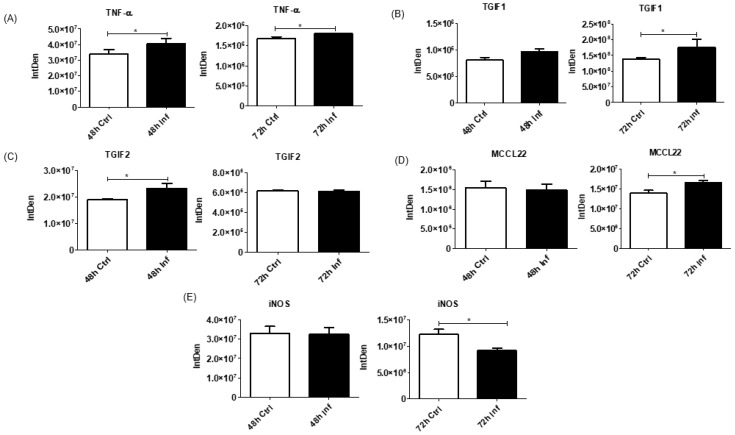
Activation of immune response after CCoV Infection. Graphs showing densitometric analysis of dot blot of (**A**) TNF-α level at 48 h and 72 h (Supplementary Figure S2L), (**B**) TGIF-1 level at 48 h and 72 h (Supplementary Figure S2M), (**C**) TGIF-2 level at 48 h and 72 h (Supplementary Figure S2N), (**D**) MCCL22 level at 48 h and 72 h (Supplementary Figure S2O), and (**E**) iNOS level at 48 h and 72 h (Supplementary Figure S2P), in isolated control and infection-derived EVs. Statistical analysis of obtained data points was performed using one-way ANOVA with Tukey post hoc analysis. Statistical significance is indicated by the mean ± SD and is defined as *p* ≤ 0.05 (*).

Different inflammatory and immune responses after CCoV infection were evaluated in the isolated EVs for various immune proteins such as IL-6 (Supplementary Figure S3A), TNF-α (Supplementary Figure S2L), IRF4 (Supplementary Figure S3B), IRF6 (Supplementary Figure S3C), C-X-C motif chemokine ligand 2 (CXCL2) (Supplementary Figure S3D), transforming growth-interacting factor (TGIF)-1 (Supplementary Figure S2M), and TGIF-2 (Supplementary Figure S2N). While IL-6, IRF4, IRF6, and CXCL2 were significantly unchanged (Supplementary Figure S3), TNF-α was significantly elevated in the infection-derived EVs at both 48 h and 72 h when compared to the control-derived EVs (* *p* = 0.01 and * *p* = 0.02, respectively) (Supplementary Figure S2L and Figure 7A, respectively). Similarly, TGIF-1 and TGIF-2 were also expressed at significantly upregulated levels at 72 h (* *p* = 0.012) (Supplementary Figure S2M and Figure 7B, respectively) and 48 h (* *p* = 0.049) (Supplementary Figure S2N and Figure 7C, respectively) after CCoV infection, respectively. We further examined the expression of a macrophage-derived chemokine, MCCL22, and found it to be significantly upregulated at 72 h in the EVs derived after virus infection compared to the control-derived EVs (* *p* = 0.02) (Supplementary Figure S2O and Figure 7D, respectively). The study of another inflammation and immune regulator, iNOS (inducible nitric oxide synthase), resulted in reduced expression at 72 h in the infection-derived EVs compared to the control-derived EVs (* *p* = 0.025), but expression at 48 h remained unchanged within EVs (Supplementary Figure S2P and Figure 7E, respectively). Hence, we confirmed the expression and packaging of key immune response-related biomarkers in EVs derived from CRFK cells after CCoV infection.

## 4. Discussion

During viral infections of inflammation, lung, and respiratory system, EVs have been established to play a central role in infection pathogenesis and disease progression. The EVs drive intercellular cross-talk and cell-to-cell progression of diseases through harboring and transporting viral and host biomolecules such as genetic elements (DNA, mRNA, miRNA, etc.), receptors, proteins, lipids, and soluble factors, collectively known as EV cargo, to neighboring or distant recipient cells in the host. Interestingly, EVs perform this transfer of biomolecules between cells without host immune recognition. Depending on virus types and host cells, small EVs can augment or restrict the infection’s progression and spread. For instance, studies have reported the role of EV subtypes after different virus infections, such as HIV, HBV, HBC, and adenovirus, for establishing productive infection and modulating cellular responses in different host cell lines [11,13,18,21,22,23]. However, there are yet limited studies for CoVs, including for previous HCoVs, animal CoVs, and recent SARS-CoV-2. In addition, exosomes from mesenchymal stem cells (MSCs), recovered COVID-19 patients, or post-COVID-19 vaccinations have been shown to confer proinflammatory effects in critical COVID-19 patients [49,50,51]. In the present study, we evaluated the impact of CCoV infection on EV biogenesis, composition, and protein trafficking in the CRFK animal model. The CRFK cells are derived from feline epithelial kidney cells, and they were receptive to CCoV infection [33,34]. The CRFK cells were infected with CCoV at MOI of 400 IFU in an exosome-free medium and incubated for different time points. The post-incubation microscopic images revealed a lesser number of viable cells with an increase in incubation time (Supplementary Figure S1). This observation was further confirmed by MTT assay which showed a significant decrease in cell viability over time (Figure 1). This result suggests that the higher cytotoxic activity in the host due to longer virus incubation length resulted in a lower cell survival rate. The EVs were isolated from the cell-free supernatant from the uninfected CRFK cells (control EVs) and after CCoV infection (infection EVs) via a series of high-speed ultracentrifugation steps. EVs were subjected to different analyses to examine the size, concentration, morphology, and composition of CRFK-derived EVs after CCoV infection. The NTA analysis revealed that there is a significant reduction in the size of CCoV-infected CRFK-derived EVs at 48 h. However, the particle size was significantly unchanged at 72 h when compared with control EVs (Figure 2C). Nonetheless, the particle size and distribution of isolated EVs from control CRFK cells and CCoV-infected CRFK cells are within the actual size range of the smallest subtype of EVs, the exosome (ranges between 30 and 200 nm), which is our primary focus in the study (Figure 2B). The negligible increase in particles per mL and total DNA and RNA levels over time and the significant increase in total protein levels in EVs derived after CCoV infection as compared to control-derived EVs could indicate the increase in EV production, packaging, and release after coronavirus infection (Figure 2D,F). Further detailed analysis of microRNAs or proteomics is warranted. TEM analysis also confirmed the production and release of EVs from the CRFK cells after CCoV infection (Figure 2A). For certain viruses, entry, spread, and infection in the host cells depend on the susceptibility and permissiveness of the host cells and/or tissues [33,52]. To explore host susceptibility further, we inoculated the human lung carcinoma epithelial A549 cells with CCoV at the same MOIs and observed for CPE formation every 24 h for 10 days. Interestingly, these cells were not receptive to CCoV infection and replication in vitro. Literature has reported that A549 expresses a negligible amount of ACE2 and no TMPRSS2, which are both established host receptors for the S protein of SARS-CoV-2 and other CoVs to enable viral entry into host cells. Hence, the transfected A549-hACE2 and A549-hACE2-TMPRSS2 that express both ACE2 and/or TMPRSS2 are generated to make them permissive to SARS-CoV-2 and other respiration infections [53]. Kumar et al. have suggested that some CoVs are resistant to in vitro cell cultures [52]. While some cells are receptive and some are not in different physiological and diseased conditions, the EVs secreted by different cell types are highly heterogeneous and cell-specific. Here, we examined the expression of several protein biomarkers such as classical markers (e.g., tumor-susceptibility gene 101 (TSG101), ALG-2-interacting protein X (Alix), CD63, CD29, E-cadherin, lysosome-associated transmembrane glycoprotein (LAMP)), biogenesis molecules (e.g., flotillin (Supplementary Figure S2B), annexin (Supplementary Figure S2C)), virus-specific proteins (syncytin-1) (Figure 3D), and inflammation and/or immune biomarkers (e.g., TLRs, HSPs, ILs (Supplementary Figure S3A), IRFs (Supplementary Figure S3B,C), caspases) within the CRFK-derived control EVs and after CCoV infection. We confirmed the presence of common and classical exosomal markers such as TSG101 (Supplementary Figure 3J), Alix (Supplementary Figure S3K), and CD63 (Figure 2E and Supplementary Figure S2A, respectively) in the EVs. TSG101 and Alix are accessory proteins of the endosomal sorting complex required for transport (ESCRT) pathway for the formation of multivesicular bodies (MVBd) during exosome biogenesis. CD63 is a tetraspanin family glycoprotein along with CD29 (integrin β1) (Supplementary Figure S3L), CD44 (a multifunctional transmembrane receptor) (Figure 4C and Supplementary Figure S2D, respectively), and LAMP (also known as CD107b) (Supplementary Figure S3E), which were also found to be expressed and hence packaged within control and infection EVs derived from CCoV-infected CRFK cells. LAMP protein, which shuttles between lysosomes, endosomes, and the plasma membrane, is a marker for lytic degranulation, and its expression within CRFK EVs indicates that CCoV is a lytic virus, similar to SARS-CoV-2, and leads to the destruction of host cells during their replication [54]. This result supports the lower cell survival rate of CRFK cells after CCoV infection which was also shown by MTT analysis (Figure 1). Other tetraspanin-associated markers including integrins (CD29) participate in cellular adhesion, motility, differentiation, metastasis, membrane fusion, and signal transduction as well as in protein trafficking. They are the common cell surface receptors for attachment and entry of exosomes and enveloped viruses such as HIV and CoVs [43,55]. Additionally, the tetraspanin-rich exosomal membrane may facilitate SARS-CoV-2 cellular entry and internalization [55]. For instance, Earnest et al. have reported that the tetraspanin-rich exosomal CD9 and TMPRSS2 interaction favored MERS-CoV entry and infection in murine lungs [56]. Another canine-specific cell–cell adhesion molecule, E-cadherin, was upregulated in infection EVs, which could indicate higher CCoV particle entry and infection in CRFK cells (Figure 4D and Supplementary Figure S2E, respectively). The calcium-dependent E-cadherin is found to promote hepatitis virus entry by regulating the membrane distribution of its receptors [57]. Flotillin is a lipid raft protein that plays key roles in many cellular processes such as endocytosis, signal transduction, cell–cell communication, autophagy regulation, and exosome subtype biogenesis. The elevated amount of flotillin-1 in the present study suggests higher protein recruitment into the raft, higher endosomal sorting, and higher EV release (Figure 4A and Supplementary Figure S2B, respectively) [41]. The annexin protein family is reported to participate in localizing mRNA, suppressing miRNA synthesis, and attenuating stress-induced dysregulation of gene expression. The members of this family can be shuttled between the cells via exosomes and from shed microvesicles. They also recruit other proteins and miRNAs into exosomes and are involved in cell–cell interactions, remodeling, and adaptive responses during diseases [42]. The reduced expression of annexin-V could suggest the suppression of apoptosis of host cells in response to stress induced by CCoV infection (Figure 4B and Supplementary Figure S2C, respectively). This phenomenon would benefit the virus via cell survival functions. We also suggest that EVs could play a critical role in the CoV transmission and extracellular virus production in the host as they have packaged and expressed significantly higher levels of host cell receptor (ACE2) (Figure 3A–C), retroviral-specific protein (syncytin-1) (Figure 3D), and commercial coronavirus protein in the infection-derived EVs at different virus incubation times. Syncytin-1 is an endogenous retroviral protein and plays a key role in cell–cell fusion and EV uptake and internalization in host cells [39,58]. The literature has documented an increased activity and expression of syncytin-1 in several diseases such as autoimmune disease, cancer, and viral diseases [59,60]. Moreover, it may function like the S protein of SARS-CoV-2. However, Lin et al. have shown a negligible similarity between them and no cross-reactivity of anti-SARS-CoV-2 spike protein antibodies with syncytin-1 in the plasma of reconvalescent COVID-19 patients [61]. TLR6 (Figure 5B and Supplementary Figure S2G, respectively) and TLR7 (Figure 5C and Supplementary Figure 2H, respectively) were significantly overexpressed in infection-derived EVs compared to control-derived EVs, while TLR3 (Figure 5A and Supplementary Figure S2F, respectively) was expressed but significantly unchanged within EVs. TLRs are pathogen recognition molecules that are localized in the cell membrane or endosomes and are expressed on most immune cells including DCs, macrophages, and natural killer (NK) cells, as well as T cells and B cells [45,46]. TLR3 and TLR6 participate in the activation of myeloid differentiation primary response 88 (MyD88), TLR7/8 activates the TIR-domain containing adaptor (TRIF, also known as TICAM1), and their collective activation causes nuclear factor-kB (NF-kβ) and IRF activation and subsequent production of type 1 IFN and proinflammatory cytokines [46]. Mazaleuskaya et al. also revealed that induction of the TLR3 pathways, not TLR2/4/7, in a mouse model with murine CoV restricts viral infection via the production of IFN-β in macrophages [44]. Hence, our result suggests that TLRs could confer a damage control effect against CCoV infection via regulating the expression of inflammatory and immune response-related biomarkers such as ILs, TNF-α, interferons, IRFs, and others. Among critical inflammation and immune response biomarkers, HSP100, TNF-α, TGIF-1, TGIF-2, mCCL22, iNOS, caspase-1, and caspase-8 were significant at different time points in the infection-derived EVs during CCoV infection of CRFK cells. While HSP70 and HSP90 are the most studied HSPs in viral infections, interestingly, HSP100 was found to be most significantly involved during CCoV infection, an indication of the usage of HSP100 chaperone machinery to replicate, adapt, and cause infection in the CRFK host cells (Figure 6A and Supplementary Figure S2I, respectively) [48]. The HSPs are molecular chaperones that check different cellular processes such as entry, replication, and survival during stressful conditions, including viral infection, and hence are very pivotal during any viral infections, including CoV infection. The NO producer iNOS is induced by cytokines and participates in host antiviral defense mechanisms, including the prevention of cytokine storms [62,63]. The lower expression of iNOS in infection EVs could suggest that CoV infection impairs the defense mechanism of the host cells and progression towards severe infection (Figure 7E and Supplementary Figure S2P, respectively). Besides host defense ability, iNOS is involved in blood flow regulation, oxygen distribution, capillary density restoration, and the prevention of cytokine storms [62]. COVID-19 studies have suggested that nitric oxide (NO) administration to COVID-19 patients could provide a beneficial effect on preventing disease progression to severity [62,63]. As other immune biomarkers, the caspase family proteins such as caspase-1 and caspase-8 were found to be at higher levels in the infection-derived EVs at 48 h when compared to control-derived EVs, which could suggest that the apoptotic pathway is initially mediated by caspase-1 (Figure 6B and Supplementary Figure S2J, respectively) and caspase-8 pathways during CCoV infection of CRFK cells (Figure 6C and Supplementary S2K, respectively). However, caspase-8 expression was found to be reduced at 72 h in infection EVs compared to control EVs, and the reduction could suggest the inhibition or delay of apoptosis and the CRFK cell survival after CoV infection over time (Figure 6C) [64]. Since caspase-1 drives the inflammation-mediated pyroptosis mechanism for non-programmed cell death, a higher level of active caspase-1 in the EVs after CCoV infection indicates inflammation and severe infection. The inflammatory caspase, caspase-1, is also required for the maturation of proinflammatory cytokines such as pro-interleukin-1B and pro-IL-18 [65]. In addition, COVID-19 studies have revealed the upregulated level of caspase-1 and its association with severe disease, longer hospitalization, and poor clinical outcomes [66,67]. Likewise, caspase-8 is involved in initiating extrinsic apoptosis to facilitate host defense via death receptors of the TNF family against pathogen infection [64]. Hence, caspase-1 and caspase-8 could trigger CRFK cell apoptosis and mediate CoV-induced inflammatory responses via cytokine release, for instance, the release of mCCL22, TNF-α, and ILs; IRF activation; and TGF-β signaling via TGIF-1 and TGIF-2 (Figure 7). Several COVID-19 studies have revealed that apoptotic caspase-1, -3, -8, and -9 are active during SARS-CoV-2 infections [64,66,67,68]. Additionally, SARS-CoV and SAR-CoV-2 viral components such as open reading frame (ORF)-3a, ORF6, ORF7a, and ORF8a have been reported to induce apoptosis of host cells via caspase-dependent pathways [69,70,71].

## 5. Conclusions

During viral infection, EVs are implicated in conferring various functions at different stages of their life cycle. They can mediate cell–cell communication, transport viral or cellular molecules, activate antiviral host defense mechanisms via immunomodulation, or assist in entry and/or during extracellular virus production in the host. Owing to the diverse functionality of EVs, they can be used as a diagnostic and therapeutic tool to suppress inflammation, cytokine storms, or multi-organ effects caused by CoVs and other virus infections. However, further work is warranted to clarify that EVs could provide a recipient-independent entry pathway for the virus and/or that the EV transfer of host or viral biomolecules is sufficient to induce inflammation, infection, and spread in the host cells. Our study has shown that the in vitro infection of CRFK cells by CCoV significantly impacts cell viability, modulates the CRFK cell derived-small EV production at the 48 h time point, and alters small EV cargo composition at varying time points in the feline cell line. However, the in vitro infection of human cell lines with animal CoVs needs further investigation which could be explored to study the cross-species host jump of animal CoVs and their adaptation to the human host. This cross-species jump has alarmed the world that future CoV pandemics are possible. Hence, to prevent future diseases caused by other CoVs, virus–host interaction in different animal hosts that are in close contact with humans should be studied thoroughly.

## Data Availability

The original data will be maintained by the corresponding author and information pertaining to the original datasets will be made available upon written request. The data presented in this study are available to open access.

## Supplementary Materials

The following supporting information can be downloaded at: https://www.mdpi.com/article/10.3390/biomedicines11030976/s1, Supplementary Figure S1. Microscopic images of control uninfected Crandell–Rees feline kidney (CRFK) cells and CRFK cells after canine coronavirus (CCoV) infection at the multiplicity of infection (MOI) of 400 infectious units (IFU) at 48 h and 72 h post-incubation. Supplementary Figure S2. Dot blot images of different EV cargo biomarkers in isolated control and infection EVs from CRFK cells after CCoV infection at 400 IFU at different time points. Supplementary Figure S3. Dot blot images of different EV cargo biomarkers in isolated control and infection EVs from CRFK cells after CCoV infection at 400 IFU at different time points.
