# Supplementary material for: Canine Coronavirus Infection Modulates the Biogenesis and Composition of Cell-Derived Extracellular Vesicles
Source: Biomedicines. 2023 Mar 21;11(3):976. doi: 10.3390/biomedicines11030976 (PMC10046050; doi:10.3390/biomedicines11030976)
Supplement: Supplementary file 1 [file biomedicines-11-00976-s001.zip › biomedicines-2136237-supplementary-Final Supplemental Figures- CCoV Manuscipt 02.24.2023.pdf]

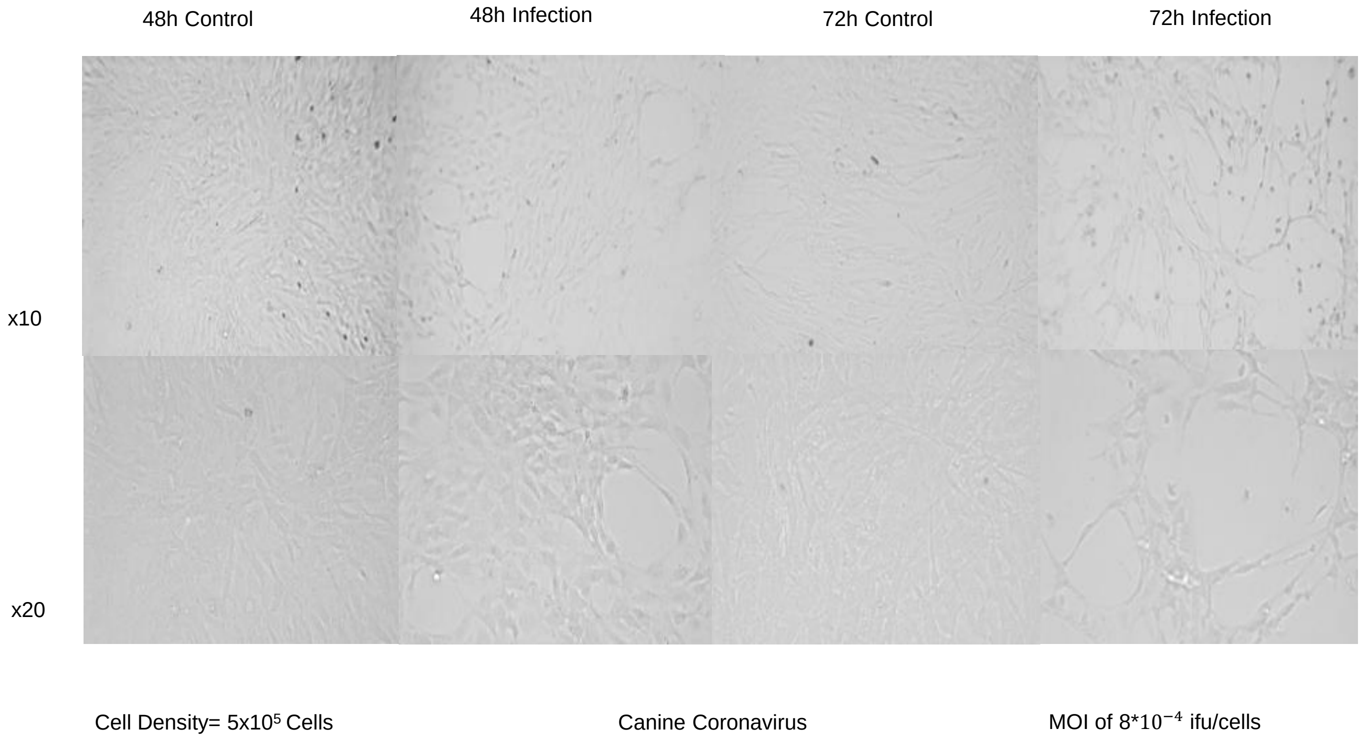

SUPPLEMENTARY FIGURE 1. Microscopic images of control uninfected Crandell-Rees Feline Kidney (CRFK) cells and after canine coronavirus (CCoV) infection at the multiplicity of infection (MOI) of 400 infectious unit (IFU) at 48h and 72h post-incubation.

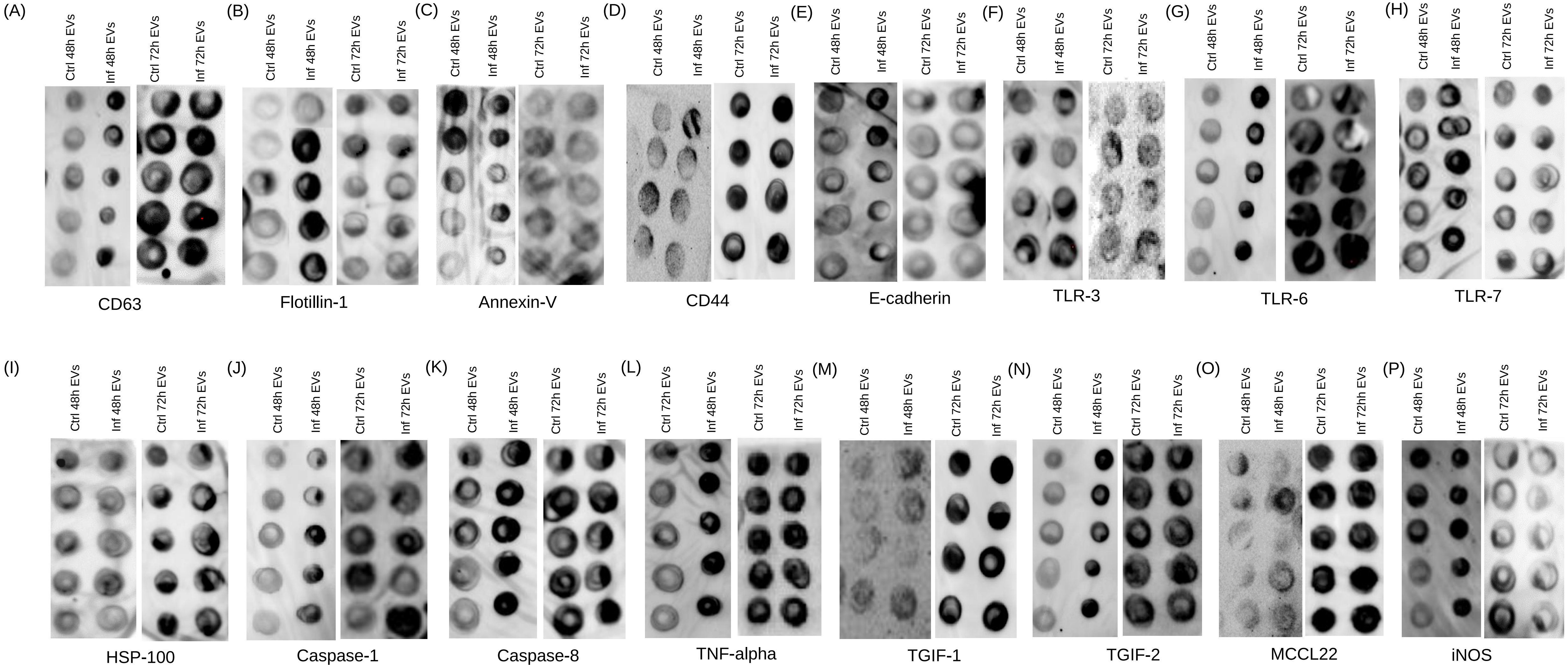

SUPPLEMENTARY FIGURE 2. Dot blot images of different EV cargo biomarkers in isolated control and infection EVs from CRFK cells after CCoV infection at 400 IFU at different time points.

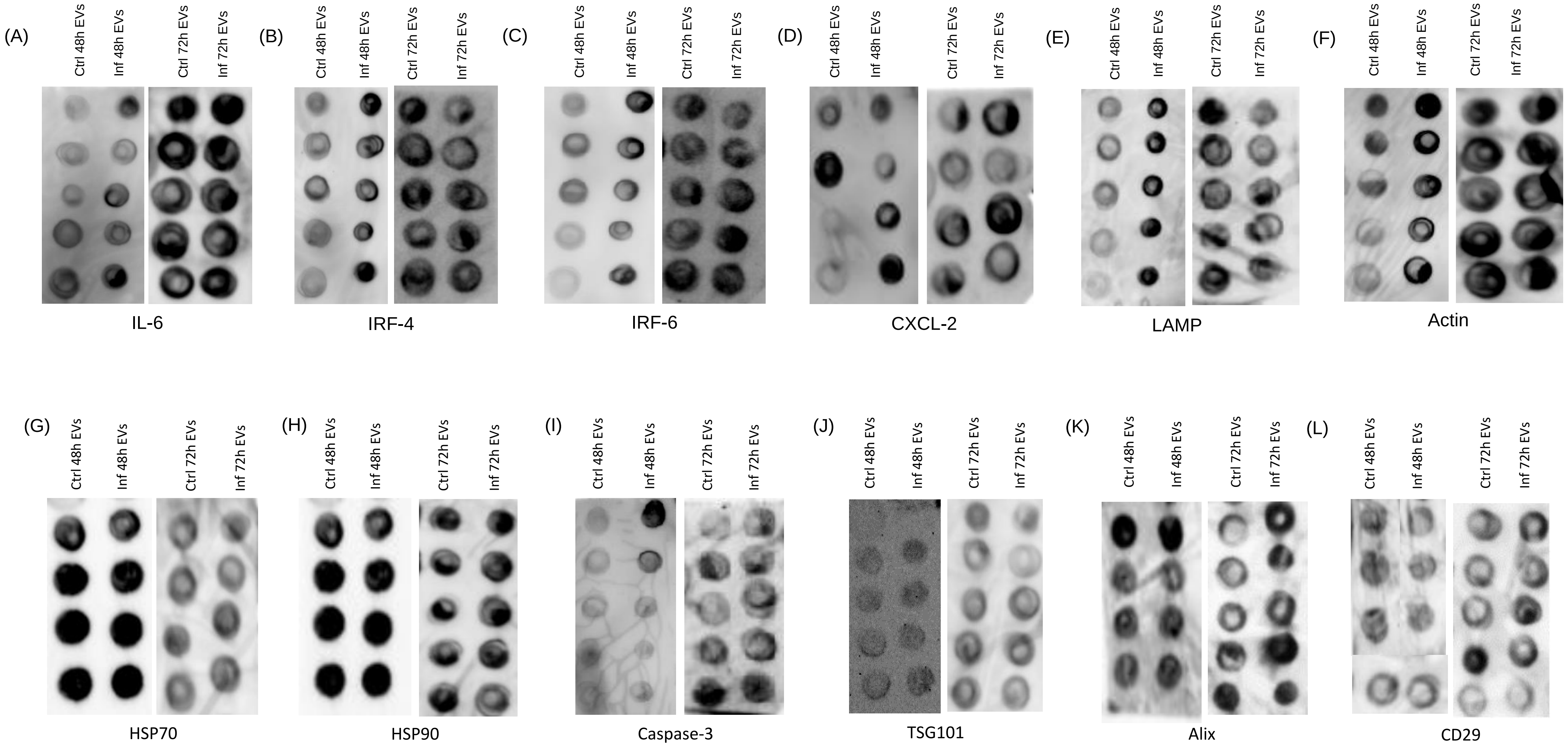

SUPPLEMENTARY FIGURE 3. Dot blot images of different EV cargo biomarkers in isolated control and infection EVs from CRFK cells after CCoV infection at 400 IFU at different time points.
